# Supplementary material for: Tamoxifen-resistant breast cancer cells exhibit reactivity with Wisteria floribunda agglutinin
Source: PLoS One. 2022 Aug 25;17(8):e0273513. doi: 10.1371/journal.pone.0273513 (PMC9409572; doi:10.1371/journal.pone.0273513)
Supplement: S1 Fig — Cell viability of T47D, T47D-TAMR, ZR75-1 and ZR75-1-TAMR cells after 72 h of TAM treatment. The LD50 of T47D and T47D-TAMR cells was 4.0 μM and 6.3 μM, respectively, and that of ZR75-1 and ZR75-1-TAMR cells was 9.5 μM and 10.4 μM, respectively. Error bars indicate standard deviations. (PDF) [file pone.0273513.s001.pdf]

S1 Fig. Cell proliferation assays in T47D, ZR75-1 and tamoxifen (TAM)-resistant cells.

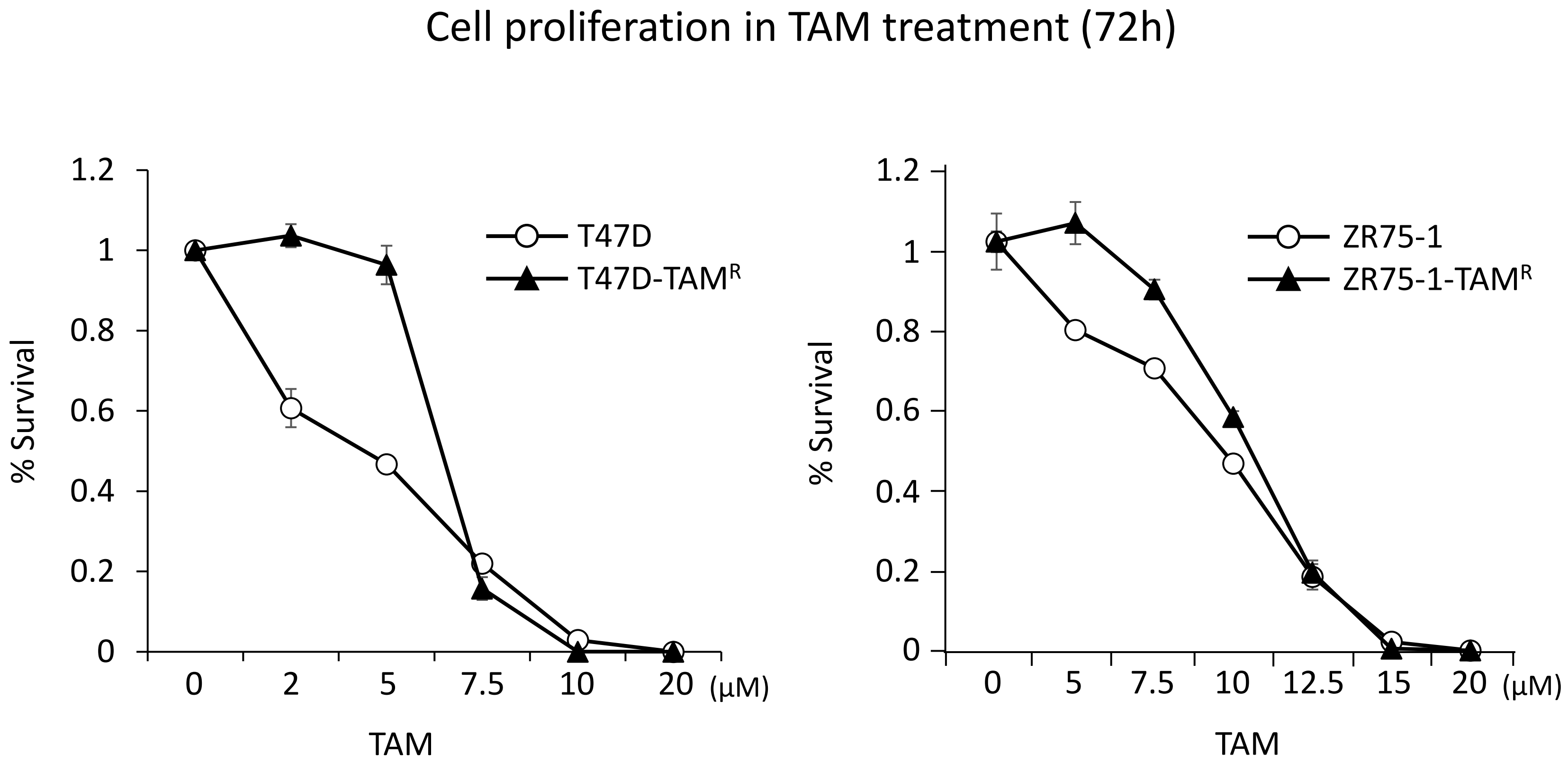

Cell viability of T47D, T47D-TAM^R, ZR75-1 and ZR75-1-TAM^R cells after 72 h of TAM treatment. The LD50 of parent T47D and T47D-TAM^R cells was 4.0 μM and 6.3 μM, respectively, and that of parent ZR75-1 and ZR75-1-TAM^R cells was 9.5 μM and 10.4 μM, respectively. Error bars indicate standard deviations.
