# Supplementary material for: Tamoxifen-resistant breast cancer cells exhibit reactivity with Wisteria floribunda agglutinin
Source: PLoS One. 2022 Aug 25;17(8):e0273513. doi: 10.1371/journal.pone.0273513 (PMC9409572; doi:10.1371/journal.pone.0273513)
Supplement: S2 Fig — WFA staining was assessed as negative, weak positive or strongly positive. Strong staining was observed mainly in the cell membrane and cytoplasm of HR-positive breast cancer tissues. (PDF) [file pone.0273513.s002.pdf]

S2 Fig. Representative images of hormone receptor-positive breast cancer tissues stained with WFA.

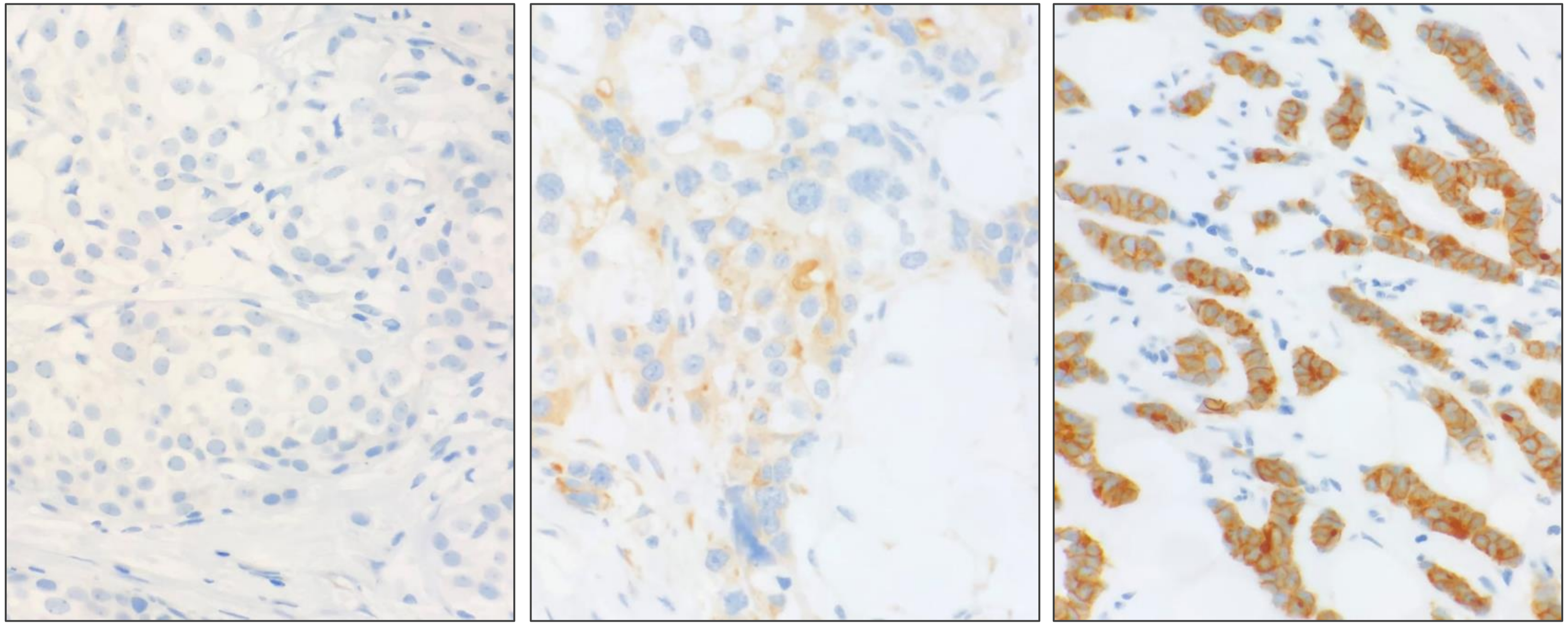

Negative Weak positive Strongly positive

WFA staining was assessed as negative, weak positive or strongly positive. Strong staining was observed mainly in the cell membrane and cytoplasm of hormone receptor-positive breast cancer tissues.
