# Supplementary material for: Tamoxifen-resistant breast cancer cells exhibit reactivity with Wisteria floribunda agglutinin
Source: PLoS One. 2022 Aug 25;17(8):e0273513. doi: 10.1371/journal.pone.0273513 (PMC9409572; doi:10.1371/journal.pone.0273513)
Supplement: S3 Fig — Lectin microarray analysis of (A) T47D and T47D-TAMR cells and (B) ZR75-1 and ZR75-1-TAMR cells, showing the relative intensities of 45 lectins in the TAM-sensitive and resistant cells, based on normalized average data. Error bars indicate standard deviations. (PDF) [file pone.0273513.s003.pdf]

S3 Fig. Lectin microarray analysis.

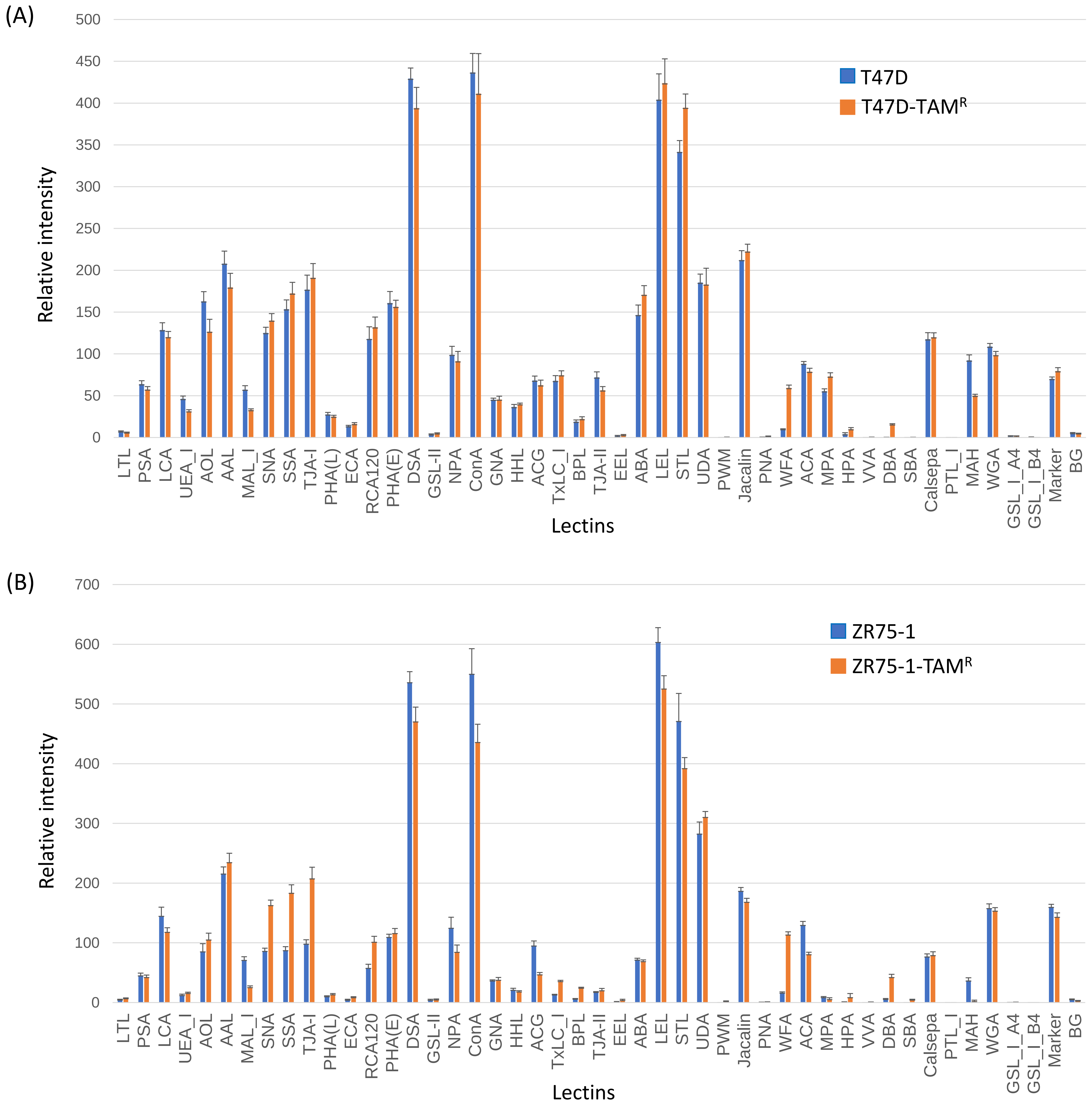

Lectin microarray analysis of (A) T47D and T47D-TAM^R cells and (B) ZR75-1 and ZR75-1-TAM^R cells, showing the relative intensities of 45 lectins in the parent and resistant cells, based on normalized average data. Error bars indicate standard deviations.
