# Supplementary material for: Tamoxifen-resistant breast cancer cells exhibit reactivity with Wisteria floribunda agglutinin
Source: PLoS One. 2022 Aug 25;17(8):e0273513. doi: 10.1371/journal.pone.0273513 (PMC9409572; doi:10.1371/journal.pone.0273513)
Supplement: S4 Fig — WFA staining of primary and metastatic lesions in three patients are shown. Histological type of patient 2 was mucinous carcinoma. Metastatic lesions of patient 1, 2 and 3 are duodenum, lung, and liver, respectively. (PDF) [file pone.0273513.s004.pdf]

S4 Fig. Comparison of WFA staining in primary and metastatic lesions.

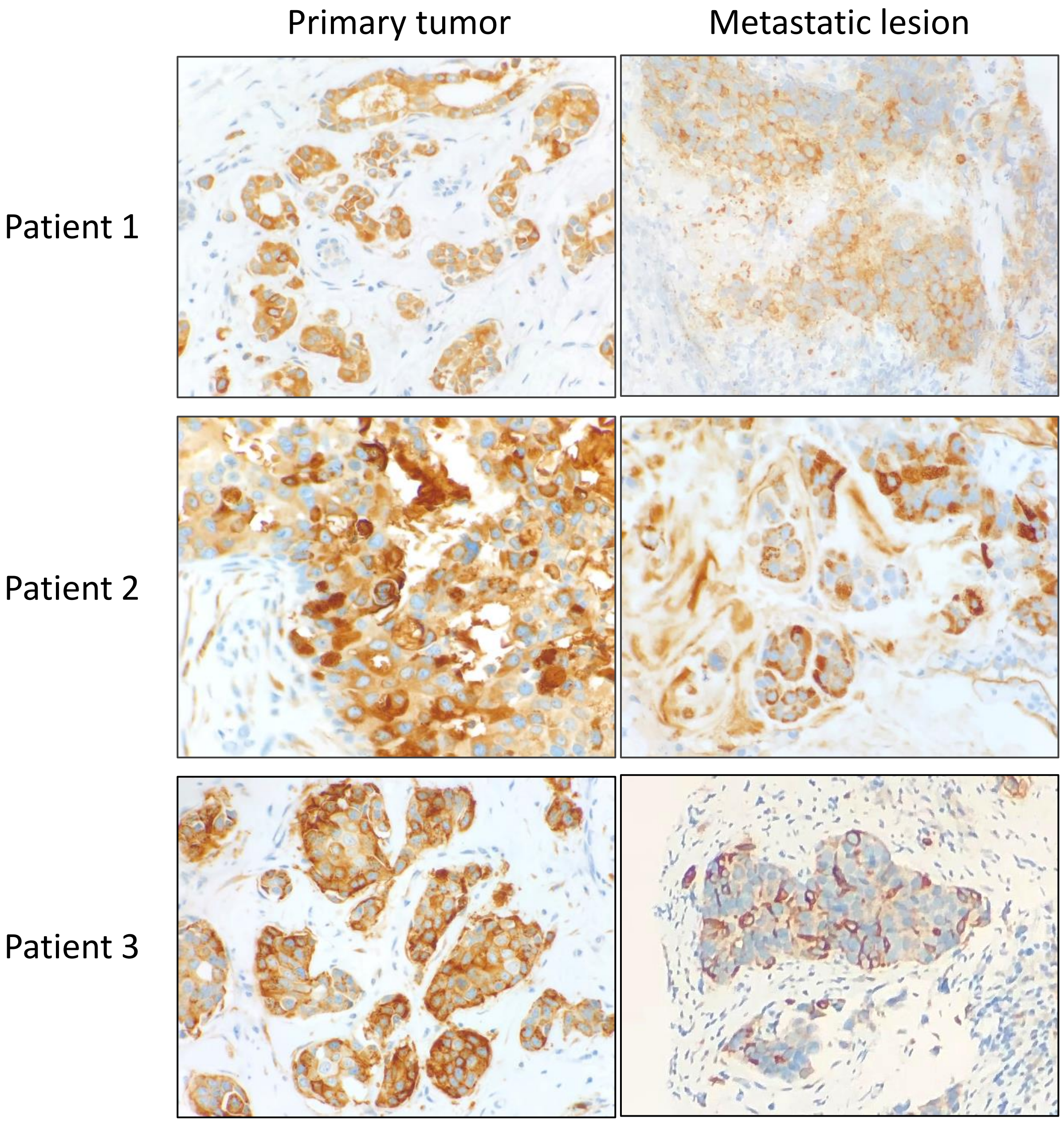

WFA staining of primary and metastatic lesions in three patients are shown. Histological type of patient 2 was mucinous carcinoma. Metastatic lesions of patient 1, 2 and 3 are duodenum, lung, and liver respectively.
