# Supplementary material for: Tamoxifen-resistant breast cancer cells exhibit reactivity with Wisteria floribunda agglutinin
Source: PLoS One. 2022 Aug 25;17(8):e0273513. doi: 10.1371/journal.pone.0273513 (PMC9409572; doi:10.1371/journal.pone.0273513)
Supplement: S5 Fig — The curve shown in red represents recurrence-free-survival of patients with B3GALNT2 mRNA-high tumors, while the black curve shows those with low B3GALNT2 tumors. The data was obtained from publicly available Kaplan–Meier plotter mRNA microarray databases [29]. (PPTX) [file pone.0273513.s005.pptx]

## Slide 1
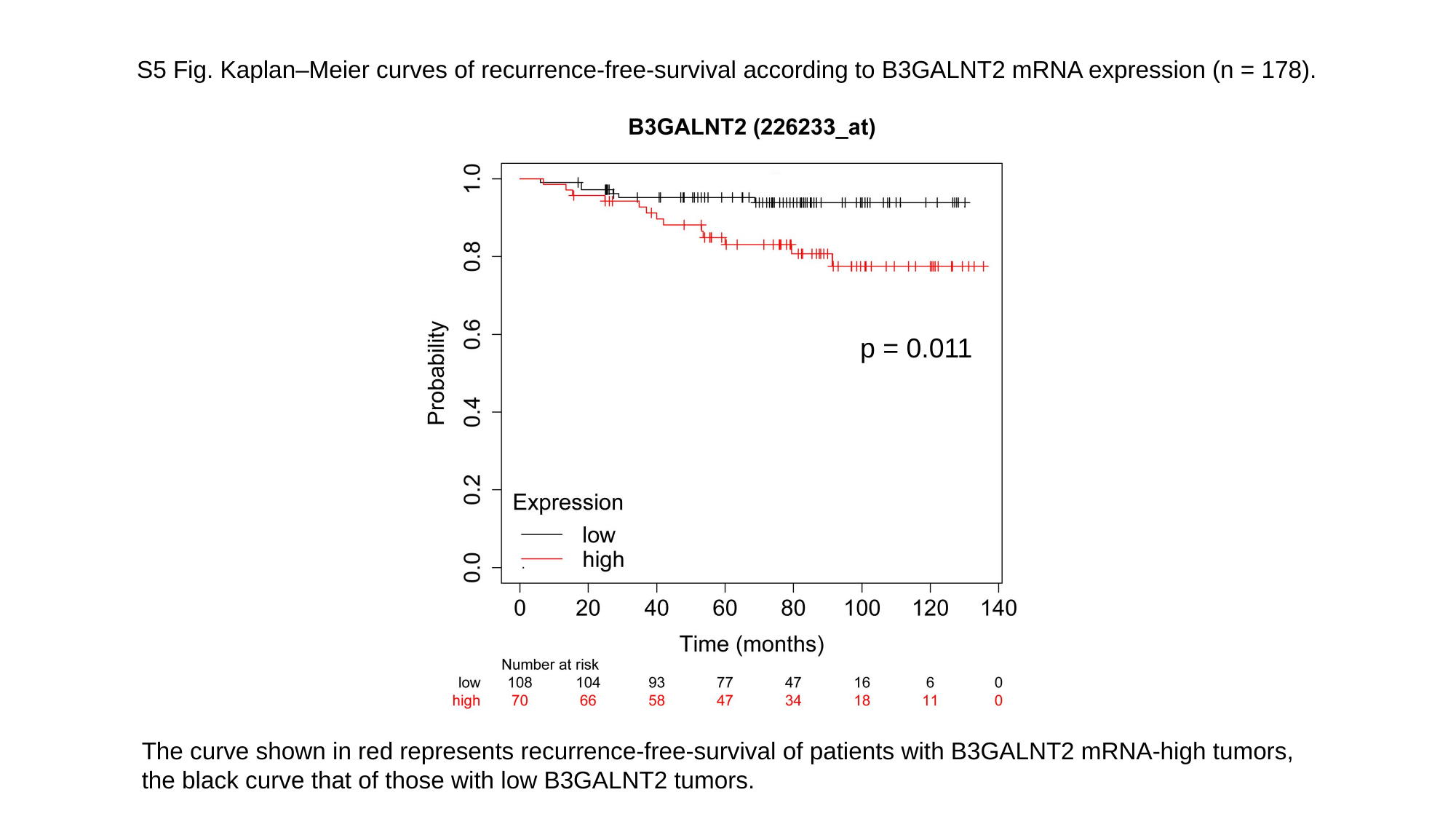

S5 Fig. Kaplan–Meier curves of recurrence-free-survival according to B3GALNT2 mRNA expression (n = 178).
p = 0.011
The curve shown in red represents recurrence-free-survival of patients with B3GALNT2 mRNA-high tumors, the black curve that of those with low B3GALNT2 tumors.
