# Supplementary figures and images for: Tamoxifen-resistant breast cancer cells exhibit reactivity with Wisteria floribunda agglutinin
Source: PLoS One. 2022 Aug 25;17(8):e0273513. doi: 10.1371/journal.pone.0273513 (PMC9409572; doi:10.1371/journal.pone.0273513)

Fig2A

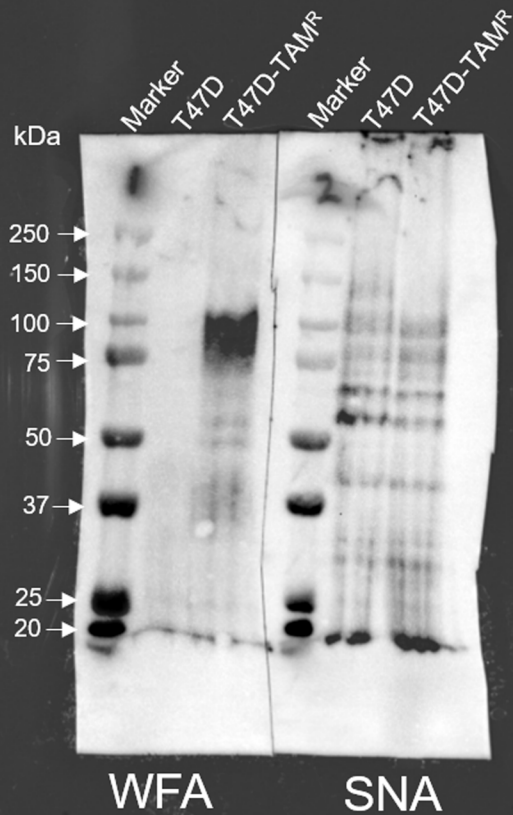

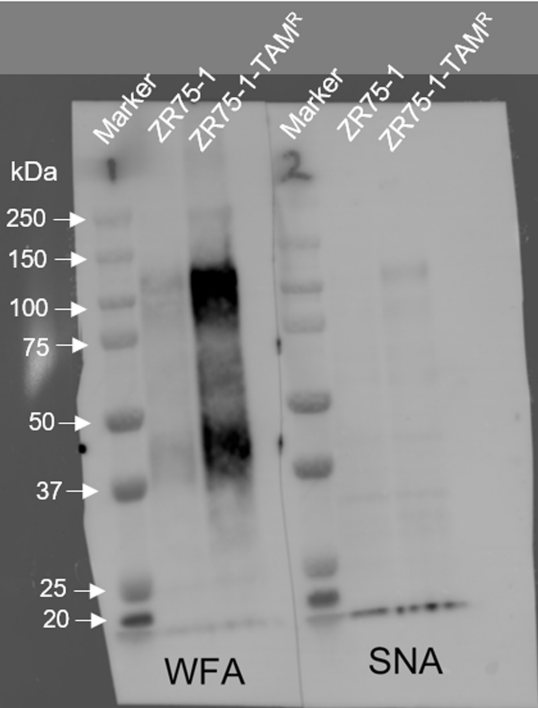

Fig2B

Fig3A

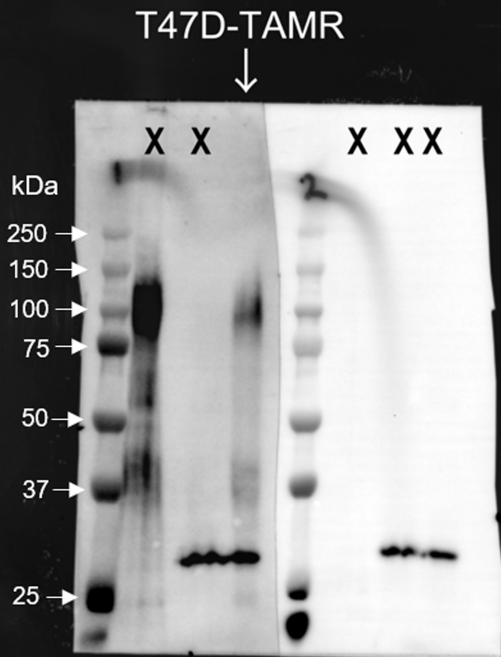

Fig3A

ZR75-1-TAMR

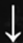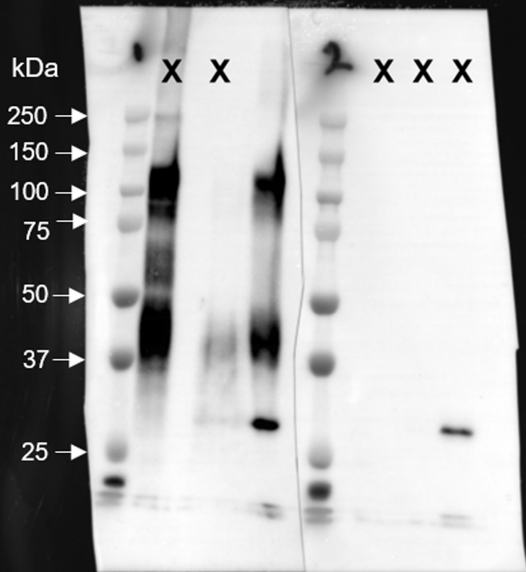

Fig3B

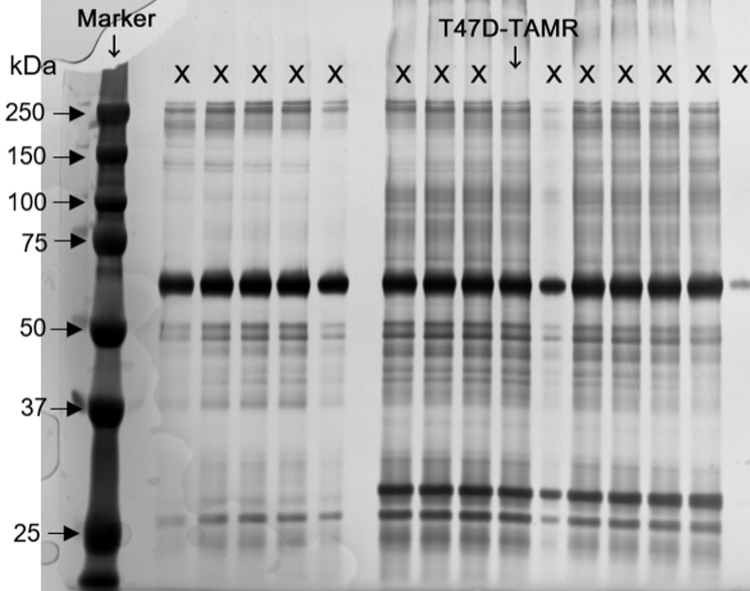

Fig3B

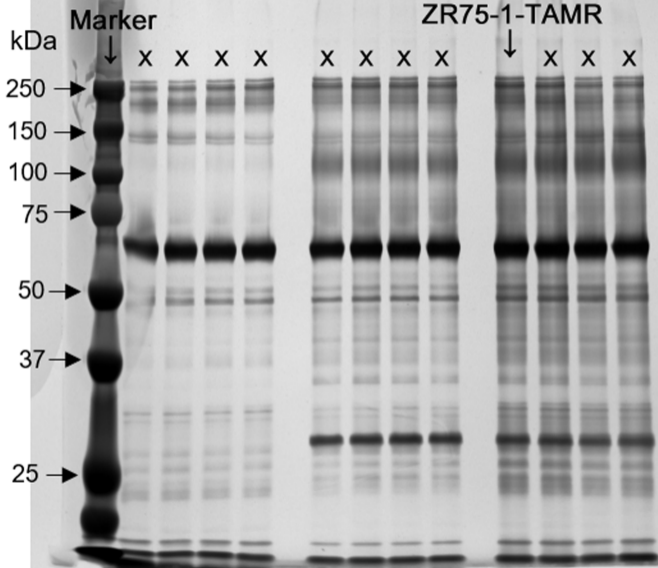

Supplement: S1 Raw images — (PDF) [file pone.0273513.s006.pdf]
